# State of HIV research in Venezuela: a systematic review

**DOI:** 10.1186/s12879-025-11654-3

**Published:** 2025-10-14

**Authors:** Jesús A. Morgado, María G. Medina, Rafael N. Guevara, Martín Carballo, Jaime R. Torres, Fhabián S. Carrión-Nessi, David A. Forero-Peña

**Affiliations:** 1 Biomedical Research and Therapeutic Vaccines Institute, Ciudad Bolívar, Venezuela; 2 “Luis Razetti” School of Medicine, Universidad Central de Venezuela, Caracas, Venezuela; 3 Department of Infectious Diseases, Hospital Universitario de Caracas, Caracas, Venezuela; 4 Instituto de Medicina Tropical “Dr. Félix Pifano”, Universidad Central de Venezuela, Caracas, Venezuela

**Keywords:** HIV, Systematic Review, Venezuela, Research gaps

## Abstract

**Background:**

Venezuela’s recent economic and political instability has severely compromised its healthcare infrastructure, impacting the management of infectious diseases, including human immunodeficiency virus (HIV). This disruption hinders progress towards the Joint United Nations Programme on HIV/AIDS (UNAIDS) 95-95-95 targets and potentially impedes HIV-related research. The current landscape of HIV research within Venezuela remains poorly characterized.

**Methods:**

A systematic review, adhering to PRISMA 2020 guidelines, was conducted using PubMed, Scopus, and *Biblioteca Virtual en Salud* databases. Original research articles pertaining to HIV in Venezuela, published between January 2003 and August 2023, were included. Commentaries, editorials, narrative reviews, and case reports were excluded. Data on study characteristics and key findings were extracted and synthesized to characterize the research landscape.

**Results:**

From 683 identified articles, 101 met the inclusion criteria. Thematic analysis revealed a concentration of studies focusing on clinical manifestations (50%), followed by epidemiological characterizations (14%), antiretroviral therapy (ART) and pediatric populations (11%). Notably, research on HIV in pregnancy was limited (4%). Epidemiological studies were often restricted to specific subpopulations, and clinical studies frequently exhibited methodological limitations, including small sample sizes and single-center designs, limiting generalizability. Conversely, research on HIV in Venezuelan migrants has increased in the past five years. Significant knowledge gaps were identified in the epidemiology of infection, ART efficacy and resistance, clinical aspects (including co-infections and opportunistic infections), and HIV in pregnancy.

**Conclusion:**

This systematic review provides a comprehensive overview of HIV research in Venezuela over the past two decades, revealing significant research gaps and potentially outdated research priorities. The paucity of comprehensive scientific production hinders accurate assessment of progress towards UNAIDS targets. Targeted research initiatives and increased investment are critical to address these knowledge gaps and improve HIV management within Venezuela.

**Supplementary Information:**

The online version contains supplementary material available at 10.1186/s12879-025-11654-3.

## Background

By the end of 2022, approximately 2.5 million individuals in Latin America were living with human immunodeficiency virus (HIV), with 120,000 new infections and 35,000 HIV-related deaths reported [1, 2]. While global HIV mortality declined by 52% by 2019, Latin America experienced a more modest reduction of 28% [3]. According to 2023 World Health Organization data, Venezuela accounts for an estimated 100,000 people living with HIV (PLHIV), with 6,062 new cases and 1,500 deaths reported in 2022 [4, 5]. Isolated cases of HIV-2 infection have also been documented [6].

Since 2016, Venezuela has faced the highest rate of antiretroviral therapy (ART) interruptions in Latin America. The situation deteriorated significantly in 2017 and 2018, with ART coverage dropping to 16% by April 2018 due to limited access [7]. This crisis precipitated a substantial emigration of Venezuelan PLHIV, placing strain on the health systems of receiving countries [8–10]. To date, the precise number of Venezuelan PLHIV who have migrated remains unquantified. In 2019, ART access was partially restored through a dolutegravir-based regimen, funded by the World Bank’s “Master Plan for Strengthening the Response to HIV, Tuberculosis, and Malaria” [11].

However, Venezuela’s ongoing economic, political, and health crisis has not only disrupted HIV care but also impeded research development [12]. Despite increased science and technology funding until 2014 [13], subsequent years have seen a decline in Venezuelan scientific output. The emigration of 16% of researchers [14], coupled with reduced funding, has significantly diminished health research capacity. Budgetary constraints have forced some state-dependent scientific institutions to rely on private investments [15, 16].

The impact of this health crisis on the HIV research landscape in Venezuela remains poorly characterized. This study was guided by the primary research question: What is the state of HIV research in Venezuela over the past two decades, and how has it been affected by the country’s protracted crisis? We hypothesized that scientific output on HIV within Venezuela has significantly declined in recent years, leading to critical knowledge gaps, while research on Venezuelan migrants with HIV conducted abroad has increased. Therefore, the primary objective of this systematic review is to characterize the body of original scientific studies on HIV/AIDS in Venezuela published between 2003 and 2023. Secondary objectives are to (1) thematically categorize the research, (2) summarize key findings across domains, and (3) identify significant, unaddressed knowledge gaps to guide future research priorities and public health policy.

## Methods

### Study design and search strategy

A systematic review was conducted in accordance with the PRISMA guidelines [17]. A comprehensive search strategy, without language restrictions, was implemented to identify relevant studies in the PubMed, Scopus, and *Biblioteca Virtual en Salud* databases. The search terms were applied to the title, abstract, and keyword fields in each database to optimize the balance between sensitivity and specificity. The search encompassed publications from January 1, 2003, to August 20, 2023. Search terms included descriptors from the DeCS (“Venezuela”, “venezolanos”, “VIH”, “Sida”) and MeSH (“Venezuela”, “Venezuelan”, “HIV”, “AIDS”). The plural term “venezolanos” was used as it is the standard descriptor for populations in Spanish-language scientific literature (e.g., “estudio en pacientes venezolanos”). An exploratory search using the singular “venezolano” was also conducted during the revision process, which confirmed that no additional relevant articles were missed by our original strategy. The detailed search strategy is provided in Supplementary Data 1.

### Eligibility criteria

Studies were included if they presented original research data on HIV/AIDS in Venezuela. This encompassed studies conducted on PLHIV as well as epidemiological investigations reporting on HIV prevalence, incidence, or risk factors within Venezuelan populations (e.g., general population, pregnant women, blood donors). Studies focusing on HIV-related knowledge or attitudes in uninfected populations, as well as basic science research not involving samples from PLHIV, were excluded. Commentaries, editorials, narrative or systematic reviews, case reports, and letters to the editor (except those containing original data) were also excluded. Furthermore, to ensure that the review synthesized data exclusively from peer-reviewed sources, articles published on preprint servers (e.g., medRxiv, bioRxiv) were not included.

### Study screening and selection

All identified citations were imported into EndNote X9, and duplicate entries were removed. Title and abstract screening was performed to assess potential eligibility for full-text review. During this initial screening of titles and abstracts, records were excluded if they were clearly irrelevant to the topic (e.g., focused on different diseases or non-Venezuelan populations), or if they were clearly of an excluded study type. Full-text articles were then evaluated against the established inclusion and exclusion criteria. This process was conducted independently by two reviewers (JAM and MGM), with any discrepancies resolved through consensus with a third reviewer (DAFP). Full texts of potentially eligible articles were retrieved, and any disagreements regarding inclusion were resolved through consensus.

### Data extraction and analysis

Data extraction included the following variables: year of publication, study design, sample size, geographical location (region or city), and key findings. Studies were categorized based on their primary content into seven thematic areas: clinical behavior, epidemiology, pediatric populations, migration, treatment (ART), HIV in pregnancy, and miscellaneous (studies not fitting into the other categories). A comprehensive list of all included studies and their key characteristics is provided in Supplementary Data 2.

## Results

**Fig. 1:**
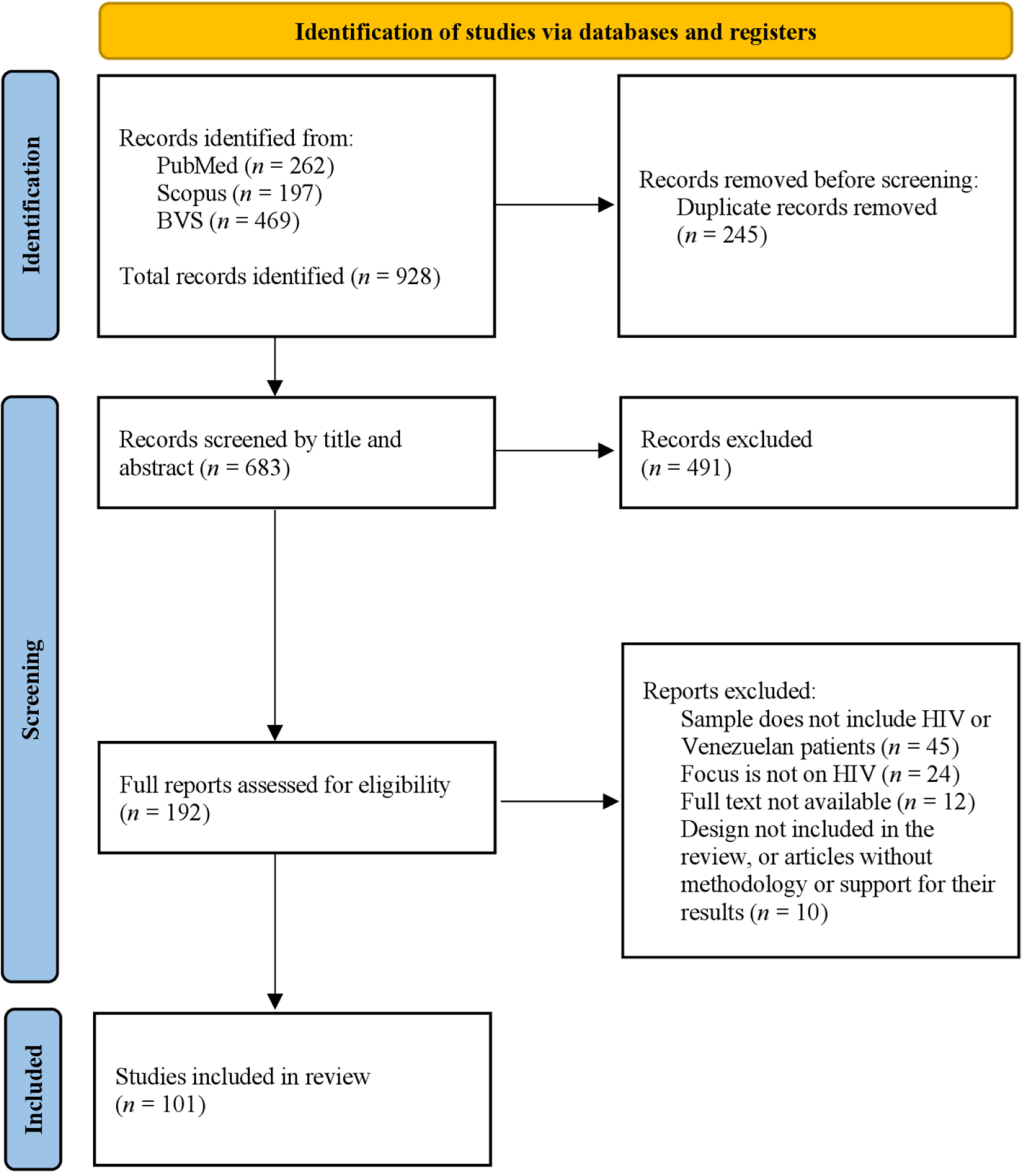
PRISMA flow diagram for systematic reviews

**Fig. 2:**
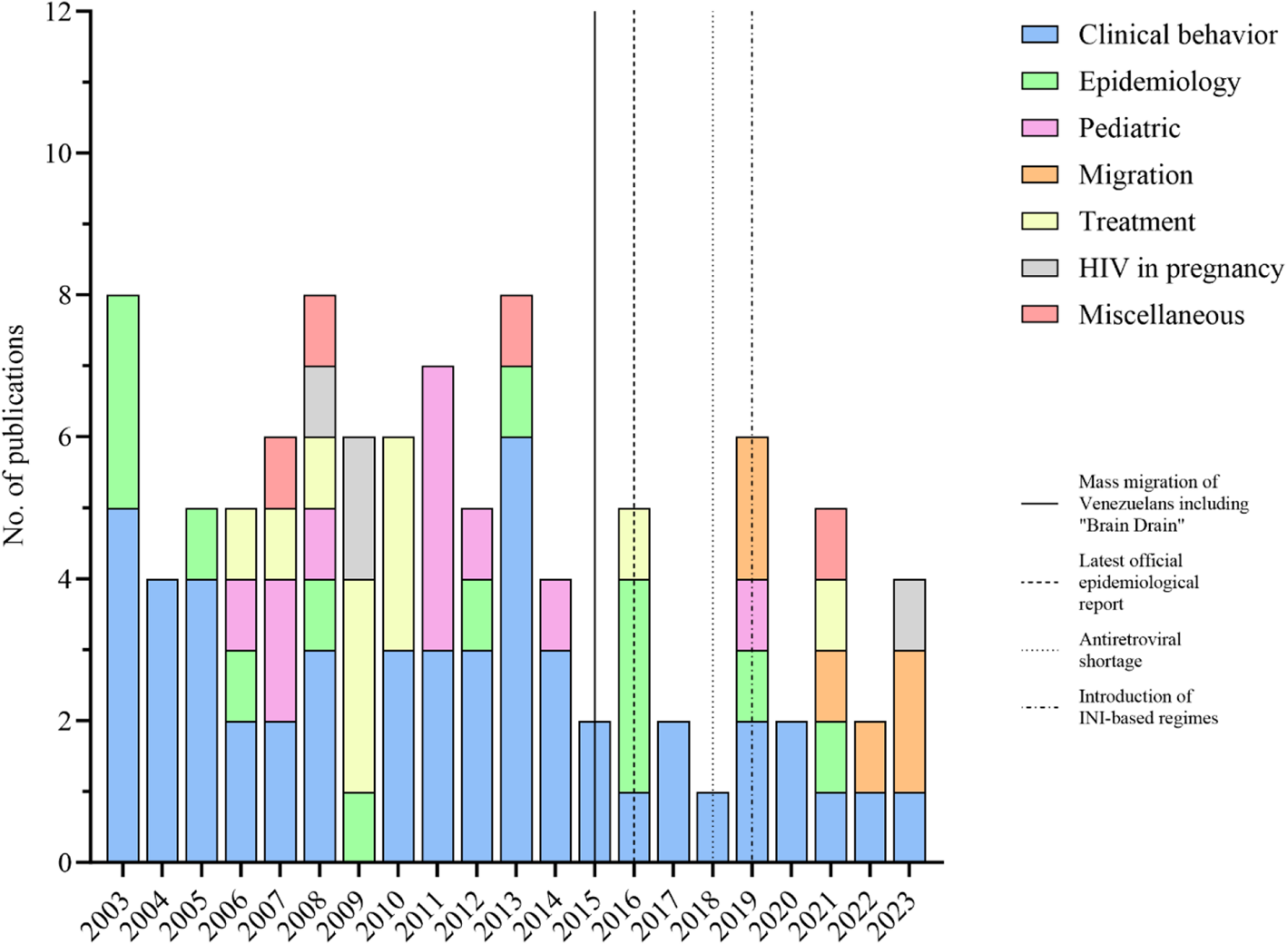
Stacked bar chart on the different types of studies analyzed by year. From “Brief communication: gaps and opportunities in HIV research in Venezuela”, by Morgado JA, et al., 2025, *AIDS *Research and Therapy, 22(1):5 [18]. Licensed under CC BY 4.0

A total of 928 records were identified in the database, with 683 remaining after duplicate removal. Among these, 101 studies met the eligibility criteria and were analyzed (Fig. 1). The majority (95%) were observational studies, including 90 cross-sectional and six longitudinal studies, while only five studies employed an experimental design. Research in Venezuela predominantly focused on the clinical behavior of the disease (50.4%), followed by epidemiological studies (13.8%), pediatrics and treatment (10.8%), migrants (5.9%), and HIV in pregnancy and miscellaneous topics (3.9%) (Fig. 2).

### Epidemiology of HIV in Venezuela

#### Prevalence studies

While HIV prevalence in the general Venezuelan population remains underexplored, specific populations have been studied (Table 1). Between 2000 and 2005, annual prevalence in pregnant women ranged from 0.4% to3.1%, peaking in 2004-2005 [19]. From 2010 to 2017, blood donors exhibited an average prevalence of 0.3% [20]. During the 2020 pandemic, respiratory symptomatic patients attending a COVID-19 referral center in Caracas showed a prevalence of 4.2% [21].

**Table 1:** HIV prevalence in different Venezuelan populations

Population	Sample size (*n*)	Region	Relation F/M	Annual prevalence	Prevalence in F/M ratio	Study period	Reference
Pregnant women	140,336	Aragua state	100%F	0.4%, 0.3%, 0.1%, 0.1%, 0.5%, 3.1%	100%F	2000 to 2005	[19]
Blood donors	6,440	South of Lara state	82%M/18%F	0.2%, 0.2%, 0.2%, 0.4%, 0.2%, 0.3%, 0.2%, 0.4%		2010 to 2017	[20]
Respiratory symptomatic patients	118	Capital District	52%F/48%M	4.2%	5.2%M/3.2%F	2020	[21]
MSM	3,175	N/A	100%M	7.8% (self-reported)	100%M	2012	[22]
Sex workers	613	Nueva Esparta state	100%F	0%	100%F	2002	[23]
Sex workers	212	Miranda state	100%F	0%	100%F	2003	[24]
Prisoners	265	Penitentiary centers INOF, La Planta, Tocorón, and Tocuyito annex	100%F	0.4%	100%F	2000	[25]
Prisoners	1,773	Six penitentiary centers	100%M	4%	100%M	1998 to 2001	[26]
Warao indigenous	576	Warao indigenous of the lower Orinoco Delta	53%M/47%F	9.6%	15.6%M/2.6%F	2013	[27]
Migrants	6,221	Colombia (Bogotá, Soacha, Soledad, and Barranquilla)	65%F/34%M/1% trans or non-binary	1.1%	1.9%M/0.6%F/8.5% trans or non-binary	2022	[28]

*MSM* men who have sex with men, *N/A* not applicable, *INOF* Instituto Nacional de Observación y Orientación para Adolescentes

Information on prevalence in risk groups (men who have sex with men [MSM], sex workers, intravenous drug users, and incarcerated individuals) is limited. In 2012, self-reported prevalence among MSM was 7.8% (Table 1) [22]. A more recent study in 2022 found a prevalence of 11.1% among Venezuelan migrant MSM in Colombia (95% CI = 4.9-17.7) [28]. Among female sex workers, only three studies were identified: two between 2002 and 2003 (with no positive cases documented) [23, 24], and a third study in 2022 reporting a prevalence of 6.6% (95% CI = 1.3-7.4) among Venezuelan migrant sex workers in Colombia [28]. In the prison population, HIV prevalence was significantly higher in men than in women. Inmates with over five years of incarceration showed a prevalence of 6.2% [25, 26]. Among indigenous communities, a prevalence of 9.6% was found in 2013 in eight Warao villages in the lower Orinoco Delta, with the Usidu community being most affected (Table 1) [27]. This high prevalence was subsequently associated with risk behaviors, MSM relationships, low disease knowledge, and risky sexual practices common in these indigenous societies [29].

#### Sociodemographic characterization

Across multiple included studies, the reported prevalence of HIV was consistently shown to increase with the onset of reproductive age, peaking among individuals aged 20-39 years [21, 22, 26, 29–48]. Some studies showed a higher prevalence among individuals aged 18 to 30 [27, 49, 50]. Males were predominantly affected (60-80%) [27, 29, 30, 39–46, 48, 50–53], with most being single [41, 54, 55]. Approximately 60% of cases occurred in MSM [21, 29, 43], and the affected population often belonged to low economic strata [39, 56]. In the female population, the most affected age range was 21-30 years [39]. Additionally, it was found that the majority were engaged in domestic tasks or were unemployed [56]. Late diagnosis of HIV was frequently reported. Factors associated with a higher risk of late presentation included being a heterosexual man, having a low socioeconomic status, and advanced age [47]. Key barriers to seeking HIV screening were identified as social stigma and lack of knowledge about the disease. Conversely, populations more likely to undergo HIV testing included women, MSM, individuals with a middle or higher income, and those with a prior history of other sexually transmitted infections [22, 47, 57].

#### Epidemiological and molecular surveillance

Numerous studies have assessed the predominant HIV-1 strains circulating in Venezuela. Based on samples collected in 1998 and 1999, it was determined that subtype B accounted for 93% of HIV-1 cases in the country [58]. Nueva Esparta state showed greater intra-subtype B genetic variability compared to samples from the capital region [58]. Subsequent investigations conducted in 2005 [59], between 2003 and 2008 [42, 60], and in 2010 [61] consistently confirmed the predominance of subtype B. While sporadic cases of other non-B subtypes and recombinant forms of HIV-1 were identified, they collectively accounted for less than 1% of all cases [60]. Phylogenetic analysis of the B/F variant revealed similarities between the Venezuelan strain and others from Latin America, possibly originating in Brazil or Argentina [62]. Across Latin America, Brazil, Argentina, and Venezuela emerged as hotspots for the spread of the HIV-1 subtype B strain [63]. Additionally, a study among the Warao community found a significant prevalence of subtype B and an isolated case of subtype C [64]. In this community, strains utilizing the CXCR4 co-receptor were more frequent (96%) than in the general population (44%) [65].

### Migration of PLHIV

Since 2015, Venezuelan migration has surged, leading to an increase in exported HIV cases in countries of the region [7]. In 2018, Peru reported that 720 Venezuelans were receiving ART, with 75.7% located in the capital [10]. Between 2017 and 2019, 398 HIV cases were documented in Venezuelans residing in Lima, representing 4.4% of new cases in 2017, 20% in 2018, and 18.7% in 2019 [66]. However, the proportion of previously diagnosed individuals decreased from 85.7% in 2017 to 39.8% in 2019 due to heightened border restrictions [66]. In 2017, 63% of Venezuelan PLHIV arriving in Peru had viral suppression, compared to only 11.2% in 2019 [66]. Meanwhile, in 2022, Colombia observed an HIV prevalence of 1.1% (95% CI = 0.6-1.4) among 6,221 Venezuelan migrants, 47.9% had a prior HIV diagnosis, and 35.7% achieved viral suppression [28]. Venezuelan migration accounted for 2.3% of HIV reported cases in Colombia in 2020 [9].

Accessing health services abroad poses several challenges for migrants. In Colombia, irregular migratory status emerged as a significant barrier [28]. In Brazil, language barriers, transportation costs, and private consultation challenges hindered access. Additionally, potential adverse reactions sometimes lead to treatment abandonment [67]. Despite these obstacles, between 2015 and 2017, 43.2% of consultations in border municipalities (such as Pacaraima) were allotted to Venezuelans, including PLHIV. A higher number of HIV consultations occurred among Venezuelans than in the local population (*p* = 0.046) [8].

### Clinical behavior

Among hospitalized acquired immunodeficiency syndrome (AIDS) patients, complications primarily affect the central nervous system (26%), respiratory system (25%), gastrointestinal system (17%), and skin/mucosa (10%) [52]. A CD4^+^ T lymphocyte count ≤ 200 cells/mm^3^ and a viral load exceeding 1,000 copies/mL were identified as risk factors for malnutrition [54]. The most frequent central nervous system complications in AIDS patients included cerebral toxoplasmosis, meningeal tuberculosis, and cerebral cryptococcosis [52, 68]. The most common symptoms associated with these complications were fever, diminished muscular strength, headache, convulsions, and altered consciousness [68]. Co-infection with *Toxoplasma gondii* led to worse outcomes in tests of memory, auditory span, and executive function [69].

#### Gastrointestinal system infections

Diarrhea is a common symptom in PLHIV [50]. Studies conducted between 1997 and 2002 consistently reported that over 40% of diarrhea cases in PLHIV have a parasitic origin [31, 48, 70, 71]. Acute diarrhea was associated with *Isospora belli* and *Entamoeba histolytica*/*dispar*, while chronic diarrhea was primarily linked to *E. histolytica*/*dispar*, *Strongyloides stercoralis*, and *Cryptosporidium parvum* [70]. During the period from 2002 to 2007, three studies found a high prevalence of parasitic co-infections (50.4%, 67.8%, and 69%), mainly involving protozoa such as microsporidia [31, 32, 48, 71] and helminths [32]. The viral etiology of diarrhea in PLHIV has received limited attention. Calicivirus was found in 12% of patients studied between 1998 and 2000, although it was not significantly associated with diarrhea [35]. In contrast, cosaviruses (3.5%), astroviruses (1.4%), adenoviruses (0.7%), and noroviruses (0.7%) were identified as causative agents of diarrhea in PLHIV between 2011 and 2013 [34].

#### Respiratory system infections

Respiratory infections are significant complications for PLHIV. Between 2004 and 2008, the most common pulmonary complications with confirmed diagnoses were *Pneumocystis jirovecii* pneumonia (49%) and pulmonary tuberculosis (38%) [52]. The prevalence of *P. jirovecii* in patients hospitalized for acute respiratory infection between 2007 and 2012 was 59.6% [72], and 17.6% in 2018 [73]. Additionally, in a group of PLHIV (only 37% of whom were receiving ART), pulmonary vascular lesions were prevalent, with hyperplasia of the middle layer of blood vessels being the most frequent finding. *P. jirovecii* was detected in 50% of patients with these lesions [74].

#### Co-infections in HIV patients

A retrospective study from 2009 to 2013 evaluated 85 histories of adolescents with HIV, documenting that tuberculosis (16.4%) and syphilis (25.9%) were the most frequent co-infections [75]. Furthermore, between 2014 and 2018, MSM and pregnant women with HIV had documented prevalences of syphilis co-infection at 7.4% and 8.2% (95% CI = 4.6-14.1), respectively [45, 49]. Co-infections with other sexually transmitted infections were common among individuals with HIV. Regarding human papillomavirus, reported prevalences vary: 5% in general practice [30], 15.8% in MSM [22], and 46-61% in patients with oral lesions [45, 76]. In 2004, a sample of 87 PLHIV revealed a 13.8% prevalence of *Ehrlichia* spp. based on white coat smear [51] (Table 2).

**Table 2:** Prevalence of key opportunistic infections and co-infections in Venezuelan PLHIV

Infection/Co-infection	Prevalence	Study population	Study period	Reference
*Ehrlichia* spp.	13.8%	87 (PLHIV)	2004	[51]
HPV	61%	31 (PLHIV)	January to March 2017	[45]
HPV	30%	20 (PLHIV)	N/A	[76]
Syphilis	7.4%	186 (PLHIV)	October and November 2012	[22]
HPV	15.8%	186 (PLHIV)	October and November 2012	[22]
HSV	6.3%	186 (PLHIV)	October and November 2012	[22]
HBV	5.9%	186 (PLHIV)	October and November 2012	[22]
HBV	9.3%	54 (PWLHIV)	2014 and 2018	[49]
HCV	3.2%	63 (PWLHIV)	2014 and 2018	[49]
Syphilis	8.2%	134 (PWLHIV)	2014 and 2018	[49]
Syphilis	25.9%	27 (PLHIV)	2009 to 2013	[75]
HPV	21.3%	47 (PLHIV)	2009 to 2013	[75]
HPV	5%	140 (PLHIV)	March to October 2010	[30]
HBV	3.1% (HBsAg)/14% (anti-HBc)	418 (PLHIV)	2002 and 2011	[77]
HCV	0.7%	418 (PLHIV)	2002 and 2011	[77]
HGV	27%	255 (*naïve* HIV-1 infected people)	N/A	[78]

*HPV* human papillomavirus, *HSV* herpes simplex virus, *HBV* hepatitis B virus, *HCV* hepatitis C virus, *HGV* hepatitis G virus, *PLHIV* people living with HIV, *PWLHIV* pregnant women living with HIV, *N/A* not available

Regarding hepatitis B and C prevalence in PLHIV, analyses conducted between 2002 and 2011 revealed that HBsAg was positive in 3.1%, anti-HBc in 14%, and anti-hepatitis C virus in 0.7%. Hepatitis B virus exposure was higher in men than in women [77]. Similar results were observed in 2012, when the self-reported prevalence of hepatitis B was 5.9% in a group of MSM [22]. Between 2014 and 2018, the prevalence of HIV and hepatitis B and C was 9.3% (95% CI = 4-19.9) and 3.2% (95% CI = 0.9-10.9), respectively [49]. Occult hepatitis virus infection was studied between 2005 and 2008, revealing that 18% of HIV-positive individuals with negative HBsAg results tested positive [77]. Additionally, the prevalence of hepatitis G in PLHIV in 2014 was 27% [78] (Table 2).

#### Skin and mucosal manifestations

Numerous studies have evaluated oral manifestations in PLHIV between 1996 and 2010. The primary etiologies included oral candidiasis (17.1-52%), hairy leukoplakia (45.3%), oral leukoplakia (29.3%), aphthous ulcerations (5.3-36.6%), and herpes simplex virus (4.2-9.3%). Although candidiasis prevalence has decreased over time, reaching maximums in 2001 and minimums in 2010 (85-10.7%), the highest prevalences were found in patients in dental practices [30, 38, 43, 55, 79]. In 2016, a study found a prevalence of oral candidiasis of 21.4% [80]. Additionally, 76% of PLHIV with oral hairy leukoplakia were confirmed to have co-infection with Epstein-Barr virus (EBV) diagnosed via polymerase chain reaction [81]. Acute lymphocytic syndrome was associated with EBV co-infection but not with cytomegalovirus [82]. Finally, a higher degree of immunosuppression significantly correlated with impaired periodontal health (*p* <0.05) [83].

Regarding cutaneous manifestations in AIDS patients, they accounted for 10% of complications at a large tertiary healthcare center. The most common were Kaposi’s sarcoma (47%) and herpes zoster (40%) [52, 80]. Furthermore, a 5% prevalence of Kaposi’s sarcoma in patients with oral mucosa lesions has been described [38]. In 2018, the prevalence of Kaposi’s sarcoma at the Vargas Hospital (Capital District) was 5.9% [73]. In ulcerated skin lesions of HIV patients, *Escherichia coli* (29.6%), *Pseudomonas aeruginosa* (18.6%), *Proteus mirabilis*, *Klebsiella pneumoniae*, and coagulase-negative *Staphylococcus* (11.1%) were the most frequently isolated pathogens [84]. *Candida albicans* vaginal candidiasis was found in 20.7% of samples collected from PLHIV [55]. Other studies reported prevalences of candidiasis by non-albicans species: 8.9% in 2002 and 33.3% in 2006 [37, 85].

### Mortality

One study revealed that HIV-associated mortality increased at a rate of 9% per sexennium. From 1996 to 2001, it accounted for 1.1% of all causes of death, rising to 1.3% from 2002 to 2007. The annual lethality rate observed in 2005 in Bolívar state was 13.2% [50]. Mortality was more pronounced in the 25-35 age group, affecting men more than women. The most affected states were Capital District, Bolívar, Monagas, and La Guaira. Between 2005 and 2007, the HIV mortality rate was 2.04 per 100,000 inhabitants. *P. jirovecii* pneumonia contributed 5.8%, and malignant tumors were responsible for 4.9%, with Kaposi’s sarcoma standing out at 1% [44, 86]. Regarding mortality associated with mycosis in PLHIV from 1996 to 2013, the main contributors were *P. jirovecii* pneumonia (66.8%), other mycoses (17.9%), and candidiasis (15.3%) [86]. In 2018, 5.5% of the deaths at the Vargas Hospital occurred in PLHIV, the majority of whom were male (76.4%) with an average age of under 40 years [73].

### Prognostic factors and immune response in HIV patients

Several studies have investigated blood factors associated with prognosis in HIV patients. A study conducted between 2002 and 2003 revealed a significant link between homocysteinemia (commonly observed in PLHIV) and coronary heart disease [87]. Another study demonstrated an inverse correlation between elevated ferritin levels and low CD4^+^ T lymphocyte count, while also establishing a direct relationship with increased erythrocyte sedimentation rate. Statistically higher ferritin levels were found in deceased patients [88]. Increased expression of the Fas/FasL system in HIV patients, along with heightened Fas-induced apoptosis, was identified. Notably, there was a significant correlation between apoptosis and viral load [89]. Another study highlighted increased superoxide production and activation of the caspase-3 pathway, which positively correlates with apoptosis [90]. In patients with HIV-*Leishmania donovani* co-infection, decreased CD4^+^ T lymphocyte viability, increased monocyte cell death, syncytium formation, and intracellular parasite replication were noted. Additionally, increased expression of CXCR4, CCR5, and NF-κB led to a T_H_2-type immune response and a more severe clinical course [91].

### Pediatric studies

In the pediatric population, five studies evaluated HIV transmission modes between 1987 and 2010. Vertical transmission accounted for 70-80% of children’s HIV infections, while horizontal transmission contributed 20-30% [92–96]. Common initial manifestations in pediatric HIV patients included generalized lymphadenopathy, hepatomegaly, upper respiratory infections, splenomegaly, parotitis, and dermatitis. Disease progression was associated with single bacterial infections, chronic diarrhea, anemia, leukopenia or thrombocytopenia, and prolonged fever [92]. Additionally, malnutrition was prevalent, especially among preschool-aged patients [97].

Between 2000 and 2004, two studies investigated oral mucosal manifestations in children with HIV. Soft tissue lesions were more prevalent, with angular cheilitis, linear erythema, candidiasis, gingivitis, and herpes simplex virus being the most frequent findings [98, 99]. In terms of co-infections, pediatric patients had lower prevalences compared to adults. Specifically, oropharyngeal candidiasis (1.1%), herpes zoster (1.1%), tuberculosis (2.2%), *P. jirovecii* (0.6%), and histoplasmosis (1.7%) were observed [92]. Another study reported prevalences of 7.6% for hepatitis A and B viruses, and 3.8% for EBV, cytomegalovirus, toxoplasmosis, and tuberculosis [100]. Additionally, a third study involving 20 infants revealed that 50% had co-infections, with the most common being cytomegalovirus and toxoplasmosis (25%) and EBV (15%) [95].

Cardiac damage associated with HIV infection between 2009 and 2010 included signs of arterial hypertension, left ventricular enlargement, mild dilated cardiomyopathy, pericardial effusion, mild systolic dysfunction, diastolic function alterations, valvular changes, and mild pulmonary hypertension in affected patients [95]. The effectiveness of ART in pediatric patients was evaluated from 1993 to 2009. Mortality and hospitalization rates remained below 5% and 10%, respectively, after treatment initiation. Lack of treatment correlated with worse clinical outcomes, and 96.4% of hospitalized patients had detectable HIV viral load values, resulting in AIDS-associated complications [93]. Another study found that 6.3% of patients who initiated ART after tuberculosis vaccine administration experienced immune reconstitution syndrome, with local manifestations in 6.3% and regional manifestations in 2.1% [101].

A retrospective study evaluated 85 histories of adolescents with HIV (2009 to 2013) and found that 50% of patients presented virologic failure after 90 months of treatment, independent of the transmission mechanism, gender, or type of initial treatment [75]. Dolutegravir-based ART yielded favorable results, although efficacy data were not reported. Mild adverse effects (myalgias, arthralgias, and insomnia) occurred in 30% of the sample, with no need for treatment regimen changes [102].

### HIV infection and pregnancy

The prevalence of HIV infection among pregnant women was 0.5% between 2004 and 2005 [19]. In a cross-sectional study of 300 women living with HIV, including 52 pregnant women, early onset of sexual activity, low schooling, and non-use of condoms were factors associated with the HIV pregnant group [56]. In a study of 58 pregnant women with HIV, most of whom had low educational and economic status, low knowledge about the disease, including the route of transmission, was documented [103]. A retrospective study analyzed the history of 80 pregnant women evaluated between 1999 and 2004. It found that in 91.5% of the cases, antiretroviral prophylaxis was indicated prenatally, at the end of pregnancy, and in the newborn. However, infection was documented in 12.5% of pregnancies that did not receive prenatal prophylaxis [104]. Recently, a study of 156 pregnant women with HIV reported co-infection rates of 9.3%, 8.2%, and 3.2% with hepatitis B virus, syphilis, and hepatitis C virus, respectively [49].

### Treatment

Treatment findings are summarized in Table 3. Six studies on ART resistance were conducted using samples from both *naïve* patients and those undergoing treatment. Among treated patients, the prevalence of acquired drug resistance to nucleoside reverse transcriptase inhibitors, non-nucleoside reverse transcriptase inhibitors, and protease inhibitors ranged from 10-65%. In contrast, *naïve* patients exhibited a prevalence of around 10-11% [42, 105–108], and there were more strains in patients infected for at least nine years than in *naïve* patients or patients with a shorter time since first diagnosis (*p* = 0.02). However, the included studies did not provide detailed analyses of the frequencies of specific resistance-associated mutations. No resistance to integrase inhibitors has been reported, although no resistance studies have been conducted after 2010. In 2017, an international multicenter study revealed that ART resistance testing was unavailable in Venezuelan public health settings, making it challenging to identify resistant strains circulating in the country [109].

**Table 3:** Summary of ART studies in Venezuela

Study focus	Key findings	Study period	Reference
Drug resistance	• Primary resistance (treatment-*naïve*): 11% • High resistance (treated patients): 47% to PIs, 65% to NRTIs, and 38% to NNRTIs • Rapid multi-drug resistance: ~ 50%	2004 and 2007	[42]
Secondary drug resistance	• High secondary mutations: 86% • Resistance in treated patients: 35% to NRTIs and 12% to NNRTIs • Resistance in naïve patients: 7.7%	N/A	[105]
Drug resistance	• High polymorphisms observed • Major resistance mutations to RTIs: 10% • Molecular markers for local subtype B epidemic: I62T and V77T polymorphisms	2003 and 2008	[106]
Drug resistance	• Recombination evidence in 5/62 isolates • Resistance mutations in 4 strains (NRTIs, NNRTIs, or PIs) • Resistance levels above WHO threshold	N/A	[107]
Drug resistance	• Additional resistance mutations in 14/23 treated patients • Plasma mutations in 7/23 patients • Associated with longer infection duration (> 9 years, *p* = 0.02)	N/A	[108]
ART use	• No access to resistance testing in Bolivia, Honduras, and Venezuela • Drug shortages and interruptions at one-third of study centers • Affected countries included Venezuela, Colombia, and others	2013 and 2017	[109]
Adverse effects	• IRU development in 37.5% (12/32) of HAART responders • Vision improvement in 90% of affected eyes	1998 and 2000	[110]
Adverse effects	• Hypertriglyceridemia	November 2008 to July 2009	[40]
Adverse effects	• ADRs caused 47.5% of first-line HAART changes • Regimens linked to ADRs: 59.6% to PIs and 41.4% to zidovudine	2010 and 2013	[41]
Effectiveness of the treatment	• Significant CD4^+^ T-cell increase • 85% of patients achieved undetectable viral load for 12 months	January 2005 and June 2009	[57]
Adherence	• Tolerance to frustration/ambiguity and high motivation predicted adherence	January and March 2018	[111]
Effectiveness of the treatment	• Treatment response increased from 34% (pre-HAART) to 66% (post-HAART) • Main cause of mortality shifted from opportunistic infections (65%) to lymphoma (70%) after HAART introduction	1986 and 2011	[112]
Effectiveness of the treatment	• Superior efficacy found in zidovudine + lamivudine + nelfinavir and stavudine + nevirapine + lopinavir/ritonavir regimens	N/A	[113]
Adherence	• High adherence (>70%) was prevalent	October 2010	[30]
Adherence	• Average adherence index: 73.2 out of 85 points	March to August 2017	[39]

*ART* antiretroviral therapy, *PIs* protease Inhibitors, *NRTIs* nucleoside reverse transcriptase inhibitors, *NNRTIs* non-nucleoside reverse transcriptase inhibitors, *RTIs* reverse transcriptase inhibitors, *WHO* World Health Organization, *IRU* immune recovery uveitis, *HAART* highly active antiretroviral therapy, *ADRs* adverse drug reactions, *N/A* not available

Three studies linked adverse effects to ART. In a group of patients with ocular cytomegalovirus, autoimmune reconstitution syndrome occurred in 37.5% of cases [110]. Another study reported lipid profile alterations, including hypertriglyceridemia (52%) and hypercholesterolemia (32%), in patients treated with a combination of integrase inhibitors and boosted protease inhibitors [40]. The primary reasons for changing ART between 2010 and 2013 were adverse drug reactions, accounting for 47.5% of cases (manifesting as anemia, gastrointestinal intolerance, and hypersensitivity). The remaining 52.5% of changes were primarily due to therapy simplification or clinical, virologic, or immunologic failure. Zidovudine was the ART most associated with adverse drug reactions occurrence, followed by protease inhibitors and efavirenz [41]. Furthermore, a study found that young patients experienced a higher frequency of adverse effects, including anemia, headache, dizziness, hypertriglyceridemia, and hypercholesterolemia [57].

Adherence to ART varied among studies, with high adherence prevailing (more than 70% of the sample exhibiting good adherence) [30, 39]. However, low adherence and high dropout rates were also observed [42]. Factors such as moderate levels of frustration tolerance/ambiguity and high motivation influenced adherence behaviors [111]. In oncology patients with HIV receiving chemotherapy, those on ART demonstrated a better treatment response compared to *naïve* patients (34% vs. 66%). Moreover, the treated group exhibited reduced hepatic and hematological involvement, along with a longer life expectancy [112]. Additionally, a 2008 study demonstrated that the regimens comprising 3’-azido-3’-deoxythymidine + lamivudine + nelfinavir and stavudine + nevirapine + lopinavir/ritonavir exhibited superior efficacy in sustaining low viral loads relative to other ART combinations (*p* <0.05) [113].

### Miscellaneous

Two studies assessed knowledge about the disease in groups of PLHIV, which had previously been associated with an increased risk of late presentation of the disease. The results were variable. A significant association was found between low educational level and lack of knowledge of the disease [39, 53]. Furthermore, it has been documented that even healthcare workers have limited knowledge regarding biosafety in the care of HIV patients [114, 115]. Another study observed that as CD4^+^ T lymphocyte count decreased, the self-esteem status of individuals worsened. Additionally, the level of self-esteem was found to decrease with the passage of years since diagnosis [116].

## Discussion

This systematic literature review provides an update on reported HIV research in Venezuela, organizing and summarizing findings from the past two decades. Overall, scientific output has declined since 2017, while studies related to migration have increased in recent years (Fig. 2) [18]. Most of the identified studies are over a decade old, indicating a lack of current data across various topics. We found a limited number of studies on first-line ART efficacy, ART resistance, cardiovascular risk among PLHIV, and HIV in pregnancy. These gaps underscore the need to prioritize research for resource allocation and evidence-based health policy [117].

National HIV prevalence remains uncertain due to limited data availability. Estimates rely primarily on projections from international agencies, as the Venezuelan Ministry of Health’s epidemiological bulletin has not been updated since 2016 [118]. Prevalence varies across populations, with indigenous communities exhibiting higher rates [27, 119, 120]. However, many studies suffer from design and sample size limitations, and updated information on risk groups is scarce. Mortality data for PLHIV are also insufficient; one study estimated an average HIV mortality rate of 2.05 per 100,000 inhabitants between 2005 and 2007, lower than in neighboring countries such as Colombia [121] and Peru [122, 123].

Although clinical studies are available, many are retrospective and lack national representativeness [21, 32–34, 38, 39, 58, 65, 87, 88]. Most research is based on data collected before 2014. Studies on HIV-tuberculosis co-infection remain limited [124], and tuberculosis prevention strategies among PLHIV have not been adequately explored despite international recommendations [125]. Information on opportunistic infections is also outdated. A 2002 study analyzing 583 serum and cerebrospinal fluid samples from PLHIV found that 19.4% tested positive for fungal infections, including histoplasmosis (50.4%), cryptococcosis (42.5%), paracoccidioidomycosis (4.4%), aspergillosis (1.8%), and coccidioidomycosis (0.9%) [126]. However, subsequent studies have lacked representative samples. Pediatric research primarily describes clinical characteristics without assessing ART efficacy, while studies on pregnant women are based on small samples, limiting insights into maternal-fetal outcomes.

The national economic crisis and healthcare system collapse have profoundly impacted Venezuelan immigrants with HIV [7, 127]. Studies show that viral suppression rates significantly declined among Venezuelan migrants arriving in Peru between 2017 and 2019 [66], likely due to ART shortages in Venezuela in 2018 [128]. However, the impact of the crisis on PLHIV remaining in Venezuela remains poorly understood. ART shortages may have also contributed to increased drug resistance, highlighting the need for research on resistance to first-line ART regimens based on integrase inhibitors [129, 130].

The decline in clinical research may be attributed to several factors. First, state funding has become scarce, with research primarily dependent on donations or private investment. Additionally, Venezuela’s economic, political, and social crisis since 2015 has led to the migration of skilled researchers, with an estimated 16% decline in the research workforce [131]. Over the past decade, multicenter studies have been rare, likely due to geopolitical challenges that have restricted access to international funding. Identifying research priorities is crucial to optimizing the allocation of limited resources.

We have identified multiple areas where information on HIV in Venezuela is outdated, including epidemiology, opportunistic infections, risk groups, and the impact of HIV during pregnancy. Significant gaps exist in understanding current HIV prevalence in both the general population and high-risk groups, as well as in assessing ART effectiveness and adherence among adults and children. Data on the efficacy and resistance of integrase inhibitors—the current first-line treatment—are also insufficient. Since 2017, research interest in Venezuelan migrants has increased, reflected in a growing number of international publications.

This study has several limitations that should be considered when interpreting the findings. Due to restricted access to paid search engines, some relevant articles were excluded. Additionally, grey literature, including undergraduate and graduate theses, conference presentations, and non-peer-reviewed publications, was not considered, potentially omitting valuable insights. Furthermore, a significant limitation of the available primary literature is that many of the included studies did not report confidence intervals or other measures of statistical uncertainty for their estimates. This restricted our ability to perform a quantitative synthesis and necessitates a cautious interpretation of the point estimates presented. Finally, we did not assess the methodological quality of the included studies, necessitating cautious interpretation of the synthesized data.

## Conclusions

This systematic review reveals a significant and concerning deterioration in HIV-related research output from within Venezuela over the last decade, confirming our initial hypothesis. The existing body of evidence is largely outdated, with critical gaps in areas essential for an effective public health response. The lack of current, country-specific data on HIV prevalence, ART efficacy, and drug resistance makes it nearly impossible to accurately measure progress towards UNAIDS 95-95-95 targets or to formulate evidence-based policies. This information vacuum not only hampers domestic efforts but also has regional implications, as the migration crisis continues to strain the health systems of neighboring countries.

To address these critical deficiencies, future research efforts must be urgently and strategically directed. We recommend prioritizing the following areas: (1) National surveillance studies to establish current HIV prevalence in the general population and in key high-risk groups, including robust monitoring of ART resistance, particularly to the current first-line dolutegravir-based regimens; (2) Operational research to evaluate the effectiveness, adherence, and outcomes of current ART programs for both adults and children; and (3) Prospective cohort studies focusing on the most vulnerable populations, such as pregnant women, indigenous communities, and people with co-infections like tuberculosis, to inform targeted and effective interventions. Renewed investment in Venezuela’s health research infrastructure is imperative to rebuild local capacity and ensure a sustainable, data-driven response to the HIV epidemic.

## Supplementary Information

Additional file 1: Supplementary Data 1. Search terms. Supplementary Data 2. Characteristics of the 101 included studies

## Data Availability

All data and materials in this article are included in the manuscript.

## Abbreviations

HIV: Human immunodeficiency virus
PLHIV: People live with HIV
ART: Antiretroviral therapy
MSM: Men who have sex with men
AIDS: Acquired immunodeficiency syndrome
EBV: Epstein-barr virus
