# Supplementary material for: State of HIV research in Venezuela: a systematic review
Source: BMC Infect Dis. 2025 Oct 14;25:1309. doi: 10.1186/s12879-025-11654-3 (PMC12522308; doi:10.1186/s12879-025-11654-3)
Supplement: Supplementary file 1 — Additional file 1: Supplementary Data 1. Search terms. Supplementary Data 2. Characteristics of the 101 included studies [file 12879_2025_11654_MOESM1_ESM.docx]

**Supplementary Data 1.** Search terms

| **Databases** | **Search terms** | **Search results^*^** |
| --- | --- | --- |
| MEDLINE (PubMed) | (((((HIV) OR (VIH)) OR (SIDA)) OR (AIDS)) AND (VENEZUELA)) OR (VENEZOLANOS) | 262 |
| Scopus | (HIV OR VIH OR sida OR aids) AND (Venezuela OR venezolanos) | 197 |
| *Biblioteca Virtual en Salud* | ((VIH) OR (HIV) OR (AIDS) OR (SIDA)) AND ((Venezuela) OR (venezolanos)) | 469 |

^*^From 01/01/2003 to 20/08/2023

**Supplementary Data 2.** Characteristics of the 101 included studies

| **Study title** | **First author** | **Year** | **Study design** | **Sample size (n)** | **Geographical location** | **Primary thematic area** | **Key findings** | **Reference** |
| --- | --- | --- | --- | --- | --- | --- | --- | --- |
| Association between parasitic intestinal infections and acute or chronic diarrhoea in HIV-infected patients in Caracas, Venezuela | Arenas-Pinto A | 2003 | Cross-sectional | 304 | Caracas | Clinical behavior | · Parasite-Diarrhea Link: *Isospora belli* & *Entamoeba histolytica/dispar* linked to acute/chronic diarrhea; *Strongyloides stercoralis* & *Cryptosporidium parvum* linked to chronic diarrhea  · Eosinophilia: Associated with strongyloidiasis, giardiasis, and isosporiasis | [70] |
| Immune recovery uveitis in AIDS patients with cytomegalovirus retinitis treated with highly active antiretroviral therapy in Venezuela | Arevalo JF | 2003 | Longitudinal | 34 | Caracas | Clinical behavior | · IRU Development: 37.5% (12/32) of HAART responders developed IRU  · Clinical Spectrum: Included vitritis, macular edema, and cataracts | [110] |
| Prevalencia de hepatitis B, hepatitis C y sífilis en trabajadoras sexuales de Venezuela | Camejo MI | 2003 | Cross-sectional | 212 | Miranda | Epidemiology | · Prevalence: Syphilis 2.4%, HBsAg 3.8%, anti-HBc 13.8%  · Hepatitis B Correlates: Linked to low education and increased age  · No HIV cases were found | [25] |
| Molecular epidemiology of HIV-1 in Venezuela: high prevalence of HIV-1 subtype B and identification of a B/F recombinant infection | Castro E | 2003 | Cross-sectional | 72 | Caracas and Nueva Esparta | Epidemiology | · Subtype B Dominance: Most samples were Subtype B  · First F Subtype ID: A recombinant B/F genetic profile was identified for the first time in Venezuela | [58] |
| Isosporiasis in Venezuelan adults infected with human immunodeficiency virus: clinical characterization | Certad G | 2003 | Cross-sectional | 397 | Caracas | Clinical behavior | · Prevalence: *I. belli* identified in 14% of subjects; 98% of these had diarrhea  · Immune/Lab Markers: Associated with eosinophilia and CD4+ counts < 200 cells/mm³  · Seasonality: Infection appeared seasonal, linked to rainy months | [48] |
| SIDA y otras afecciones de transmisión sexual en presidiarias de la región central de Venezuela | Figueroa Brito J | 2003 | Cross-sectional | 265 | Aragua, Carabobo, and Miranda | Epidemiology | · Sexual Behavior: Mean age of first sexual relationship was 16.75 years; pattern was highly heterosexual (89%)  · High Disease Prevalence: Syphilis was 117/1,000 and AIDS was 377/1,000, far exceeding national rates | [27] |
| Bacterias aerobias aisladas de úlceras cutáneas en pacientes VIH-positivos: Instituto de Biomedicina sección de microbiología: años 2001-2002 | Marcano Lozada M | 2003 | Cross-sectional | 19 | Caracas | Clinical behavior | · Bacterial Profile: Isolates were 74% Gram-negative, mainly *E. coli* (29.6%) and *P. aeruginosa* (18.6%)  · Resistance: High resistance noted in *P. mirabilis* and *P. aeruginosa*; AMC-R *E.coli* was common | [84] |
| Histopathological analysis and demonstration of EBV and HIV p-24 antigen but not CMV expression in labial minor salivary glands of HIV patients affected by diffuse infiltrative lymphocytosis syndrome | Rivera H | 2003 | Cross-sectional | 7 | Caracas | Clinical behavior | · Histopathology: Showed lymphocytic infiltration, mainly periductal  · Immunohistochemistry: Positive for LMP-EBV & p-24 proteins in all cases; negative for CMV. Lymphocytes were CD8 positive | [82] |
| Morphologic identification of Ehrlichia sp. in the platelets of patients infected with the human immunodeficiency virus in Venezuela | De Tamí IDC | 2004 | Cross-sectional | 87 | Caracas | Clinical behavior | · Ehrlichia Prevalence: Found in 13.8% (12/87) of study subjects, suggesting it as a possible diagnosis in HIV-infected patients | [51] |
| Increased Fas-mediated apoptosis in polymorphonuclear cells from HIV-infected patients | Salmen S | 2004 | Experimental | 52 | Merida | Clinical behavior | · Accelerated Apoptosis: HIV+ patients showed accelerated spontaneous apoptosis and elevated susceptibility to Fas-induced apoptosis | [89] |
| Prevalence of Oral Candidiasis in HIV/AIDS Patients: A Retrospective Study | Tovar V | 2004 | Cross-sectional | 509 | Caracas | Clinical behavior | · Prevalence: 29.66% of AIDS patients had oral Candida lesions | [43] |
| Vaginal flora in HIV-positive and HIV-negative patients | Villalobos N | 2004 | Cross-sectional | 106 | Zulia | Clinical behavior | · HIV+ Patients: 66% were pregnant; most were 21-35 years old  · Main Infection: *Candida albicans* was the primary infectious agent (20.75%) in both groups | [55] |
| Trends of HIV-1 molecular epidemiology in Venezuela: introduction of subtype C and identification of a novel B/C mosaic genome | Castro E | 2005 | Cross-sectional | 106 | Caracas | Clinical behavior | · Subtype B Prevails: Clade B remains high (90.6% env, 94.3% gag)  · New Subtypes: First identification of Subtype C & B/C recombinant viruses in Venezuela | [59] |
| Cryptosporidiosis in HIV-infected Venezuelan adults is strongly associated with acute or chronic diarrhea | Certad G | 2005 | Cross-sectional | 397 | Caracas | Clinical behavior | · Prevalence: *Cryptosporidium sp.* identified in 15% of subjects  · Strong Associations: Linked to diarrhea, weight loss, and CD4+ counts < 100 cells/mm³ | [31] |
| Detection of Candida dubliniensis in Venezuela | De Capriles CH | 2005 | Cross-sectional | 402 | Caracas | Clinical behavior | · *C. dubliniensis* ID: Identified in 3.98% of HIV+ individuals  · Fluconazole Resistance: 19% of *C. dubliniensis* isolates were resistant | [85] |
| Calicivirus infection in human immunodeficiency virus seropositive children and adults | Rodríguez-Guillén L | 2005 | Cross-sectional | 240 | Caracas | Clinical behavior | · Pediatric Caliciviruses: HIV+ children were twice as likely to have caliciviruses (51% vs. 24%)  · Diarrhea Link: Viral infections were not significantly associated with diarrhea | [35] |
| The analysis of near full-length genome sequences of human immunodeficiency virus type 1 BF intersubtype recombinant viruses from Chile, Venezuela and Spain reveals their relationship to diverse lineages of recombinant viruses related to CRF12_BF | Sierra M | 2005 | Cross-sectional | 6 | Venezuela, Chile and Spain | Epidemiology | · BF Recombinant Study: Analysis of 6 BF recombinant HIV-1 genomes supported common ancestry related to CRF12_BF | [62] |
| Prevalence of oral lesions in HIV+ patients, relationship with CD4+ cell count and viral load in a Venezuelan population | Bravo IM | 2006 | Cross-sectional | 75 | Caracas | Clinical behavior | · Prevalence: 85% of HIV/AIDS patients had associated oral lesions  · Common Lesions: Oral Candidiasis (61%), Oral Hairy Leukoplakia (53%)  · Viral Load/CD4 Link: Lesions were independent of CD4 count at VL >30,000 copies/mm³; patients with CD4+ <200 cells/mm³ were more susceptible | [38] |
| High prevalence of secondary resistance mutations in Venezuelan HIV-1 isolates | Dieudonne M | 2006 | Cross-sectional | 30 | Caracas | Treatment | · Secondary Mutations: 86% of Subtype B samples had secondary mutations  · Drug Resistance: Resistance in treated patients was 35% to NRTIs and 12% to NNRTIs; resistance in naïve patients was 7.7% | [105] |
| Prevalence of intestinal microsporidiosis and its contribution to diarrhea in a group of human immunodeficiency virus-infected patients from Zulia State | Chacin-Bonilla L | 2006 | Cross-sectional | 103 | Zulia | Clinical behavior | · Prevalence: Microsporidial infections in 13.6% of patients  · Diarrhea Link: No significant association found between Microsporidia and diarrhea | [71] |
| HIV/AIDS: soft oral tissues in children aged 0-4 years 2002-2004 | Guerra ME | 2006 | Cross-sectional | 59 | Caracas | Pediatric | · Significant Oral Lesions: Statistically significant differences between HIV(+) and HIV(-) infants for Angular Cheilitis, Xerostomia, Linear Erythema, Gingivitis, & Herpes Simplex Virus | [98] |
| Assessment of human immunodeficiency virus type 1 and risk practices among female commercial sex workers in Isla Margarita, Venezuela | Munoz B | 2006 | Cross-sectional | 613 | Nueva Esparta | Epidemiology | · HIV Negativity: No FCSW tested HIV+, correlating with low self-reported STI rates  · Condom Use: Regular with clients (>80%); uncommon in non-client relations (<20%) | [24] |
| HIV type 1 drug resistance among naive patients from Venezuela | Bouchard M | 2007 | Cross-sectional | 20 | Caracas | Treatment | · RTI Resistance: 10% prevalence of major resistance mutations to RTIs  · Molecular Markers: Protease polymorphisms I62T and V77T suggested as markers for the local Subtype B epidemic | [106] |
| Level of knowledge and attitude of dentists towards HIV/AIDS carriers | Dávila ME | 2007 | Cross-sectional | 48 | Caracas | Miscellaneous | · Knowledge-Attitude Gap: 60.4% reported good knowledge, but 43.8% held negative attitudes  · Reluctance to Treat: 45.8% were reluctant to treat HIV/AIDS patients | [53] |
| Oral lesions and immune status in children with HIV/AIDS | Guerra ME | 2007 | Cross-sectional | 154 | Caracas | Pediatric | · Lesion Correlation: Oral lesions were directly related to immunological status and were more frequent with severe immunosuppression | [99] |
| Hyperhomocysteinemia in Venezuelan adults infected with human immunodeficiency virus | Martí-Carvajal A | 2007 | Cross-sectional | 80 | Carabobo | Clinical behavior | · Prevalence: 46.9% overall prevalence of hyperhomocysteinemia  · Elevated Levels: 23.4% of patients had serum homocysteine levels > 15µM | [87] |
| Mechanisms of neutrophil death in human immunodeficiency virus-infected patients: role of reactive oxygen species, caspases and map kinase pathways | Salmen S | 2007 | Experimental | 53 | Merida | Clinical behavior | · PCD Mechanism: Spontaneous neutrophil death is caspase-3 dependent  · Oxidative Stress: Accelerates neutrophil death by activating caspase-3  · p38 MAPK Role: Promotes neutrophil survival in HIV+ patients | [90] |
| Initial manifestations of human immunodeficiency virus infection in pediatrics | Siciliano Sabatela L | 2007 | Cross-sectional | 19 | Caracas | Pediatric | · Transmission: 80.1% vertical transmission  · Common Manifestations: Generalized lymphadenopathy (25%), severe bacterial infection (18.2%) | [92] |
| Infection control attitudes and perceptions among dental students in Latin America: implications for dental education | Acosta-Gío AE | 2008 | Cross-sectional | 731 | Latin America (Costa Rica, Mexico, and Venezuela) | Miscellaneous | · Deficits: Students had incomplete knowledge and lacked confidence in infection control  · Bias: Many held prejudices against infected individuals | [115] |
| Antiretroviral prophylaxis in 80 pregnant women infected with human immunodeficiency virus | Carvajal A | 2008 | Longitudinal | 80 | Caracas | HIV and pregnancy | · Demographics: Most patients (63%) were 20-29 years old  · ARV Prophylaxis: 91.5% received ARV prophylaxis  · Outcomes: Average CD4+ count was 527.6 cells/mm³; average viral load was 150.24 copies/mm³ post-prophylaxis | [104] |
| Determinants of late disease-stage presentation at diagnosis of HIV infection in Venezuela: A case-case comparison | Bonjour MA | 2008 | Cross-sectional | 225 | Carabobo | Clinical behavior | · Late Presenters: 40% (91/225) were defined as late presenters  · Risk Factors: Included older age (>30 years), male heterosexuality, and lower socio-economic status  · Barriers to Testing: Included low knowledge, lack of perceived risk, and stigma | [47] |
| Hypercalciuria is the main renal abnormality finding in Human Immunodeficiency Virus-infected children in Venezuela | Gonzalez C | 2008 | Cross-sectional | 26 | Carabobo | Pediatric | · Key Health Issues: HIV+ children were often short for age and malnourished  · Kidney Findings: Tubular disorders were common, including hypercalciuria, hyperchloremia, and acidosis | [100] |
| HIV, hepatitis B and syphilis infection in inmates of Venezuela's prisons, 1998-2001 | Posada A | 2008 | Cross-sectional | 1,773 | Tachira, Caracas, Yaracuy | Epidemiology | · Prevalence: Hepatitis B (16.2%) was highest, followed by Syphilis (6.1%) and HIV (4.0%)  · Age Trends: HIV was prevalent in the youngest inmates (18-37 yrs); Hep B increased with age | [26] |
| Human immunodeficiency virus (HIV) infection in medical services | Sandoval de Mora M | 2008 | Cross-sectional | 53 | Bolivar | Clinical behavior | Demographics: Prevalent in 22-31-year-old males  · Clinical Presentation: Fever and weight loss were common; digestive & CNS were the most affected systems | [50] |
| Viral loads in pediatric HIV patients with antirretroviral treatment | Porto-Espinoza L | 2008 | Cross-sectional | 50 | Zulia | Pediatric | · Superior Efficacy Regimens: Zidovudine + lamivudine + nelfinavir and stavudine + nevirapine + lopinavir/ritonavir were most effective in sustaining low viral loads | [113] |
| Oral health conditions in adult HIV/AIDS patients | Santana Y | 2008 | Cross-sectional | 246 | Zulia | Clinical behavior | · High Needs: 58.9% had multiple caries; 77.2% required periodontal treatment  · Common Oral Lesions: High prevalence of xerostomia (54.1%), aphthous ulcerations (36.6%), and oral candidiasis (17.1%) | [79] |
| Surveillance of HIV type 1 drug resistance among naive patients from Venezuela | Castillo J | 2009 | Cross-sectional | 65 | Zulia | Treatment | · Subtype & Resistance: Most strains were Subtype B; 4 strains had resistance mutations to NRTIs, NNRTIs, or PIs  · High Prevalence: Resistance mutations were above the 5% WHO threshold in this untreated cohort | [107] |
| Knowledge about HIV/AIDS in a group of HIV-positive pregnant women | Guerra M | 2009 | Cross-sectional | 58 | Caracas | Miscellaneous | · Knowledge Gap: Findings indicated inadequate knowledge regarding HIV transmission routes | [103] |
| Trends in the prevalence of HIV and syphilis among pregnant women under antenatal care in central Venezuela | López-Zambrano MA | 2009 | Cross-sectional | 148,554 | Caracas | HIV and pregnancy | · Syphilis Prevalence: Remained stable at 0.81%  · HIV Prevalence: Increased from 0.41% (2000) to 3.05% (2005), though not statistically significant | [19] |
| Comparative analysis of polymorphisms in the HIV type 1 pol gene in the proviral DNA and viral RNA in the peripheral compartment | Rangel HR | 2009 | Cross-sectional | 34 | Caracas | Treatment | · Compartmentalized Resistance: 14/23 treated patients showed additional resistance mutations in only one compartment  · Time-Dependent Differences: Greater differences seen in patients infected for >9 years | [108] |
| HIV diversity in Venezuela: predominance of HIV type 1 subtype B and genomic characterization of non-B variants | Rangel HR | 2009 | Cross-sectional | 425 | Caracas | Epidemiology | · Predominant Subtype: HIV-1 Subtype B accounted for 99.1% of isolates  · Non-B Variants: Four non-Subtype B isolates were found (Subtype C, AG recombinant, and two HIV-2) | [60] |
| Prevalence of antiretroviral drug resistance among treatment-naïve and treated HIV-infected patients in Venezuela | Rangel HR | 2009 | Cross-sectional | 116 | Caracas | Treatment | · Primary Resistance (naïve): 11%  · High Resistance (treated): 47% to PIs, 65% to NRTIs, and 38% to NNRTIs  · Rapid Multi-drug Resistance: ~50% of patients showed highly reduced susceptibility within five years of diagnosis | [42] |
| Detection of Candida Albicans in oral hairy leukoplakia lesions in a group of HIV+ Venezuelan patients | González X | 2010 | Cross-sectional | 21 | Caracas | Clinical behavior | · Detection Rates: *C. albicans* was found in 100% of OHL patients by Grocott stain, but only 29% by culture | [36] |
| Neurological opportunistic diseases in HIV infected patients in a Western hospital of Venezuela, 2007 - 2009 | Herrera Martínez AD | 2010 | Cross-sectional | 228 | Lara | Clinical behavior | · Primary Hospitalization Cause: Neuroinfection  · Most Common Brain Lesion: Toxoplasmosis | [68] |
| Epstein Barr Virus detection and latent membrane protein 1 in oral hairy leukoplakia in HIV+ Venezuelan patients | González X | 2010 | Cross-sectional | 21 | Caracas | Clinical behavior | · EBV Prevalence: Higher in HIV+ OHL patients (76%) vs. HIV- subjects (50%)  · LMP-1 Expression: High expression (60%) in HIV+/EBV+ OHL basal cells | [81] |
| Absence of primary integrase resistance mutations in HIV type 1-infected patients in Venezuela | Rangel HR | 2010 | Cross-sectional | 57 | Caracas | Treatment | · Low Primary Resistance: Only one non-drug-conferring primary integrase resistance mutation was found, likely due to the recent introduction of these inhibitors | [61] |
| Absence of primary integrase resistance mutations in HIV type 1-infected patients in Venezuela | Sandoval de Mora M | 2010 | Experimental | 54 | Bolivar | Treatment | · High CV Risk Factors: High rates of hypertension (31.18%), smoking (44.44%), and dyslipidemia (33.33%)  · Overall Risk: Calculated 10-year Framingham CVD risk was mostly low (68.52%) to moderate (29.63%) | [46] |
| Effect of antiretroviral therapy on the lipid profile of AIDS patients in Maracaibo, Zulia State, Venezuela | Soto I | 2010 | Cross-sectional | 50 | Zulia | Treatment | · Lipid Changes: Triglycerides, total cholesterol, and LDL-C significantly increased by the study's end on a PI/b + NRTI regimen | [40] |
| Detection of Human Papillomavirus in the gingival fluid of patients with human immunodeficiency and periodontal disease | Escalona CL | 2011 | Cross-sectional | 34 | Caracas | Clinical behavior | · HPV Detection: Found in 46% of HIV+ patients on HAART and was significantly linked to higher HIV viral loads | [76] |
| Progression of HIV infection in adolescents according to route of transmission | López MG | 2011 | Cross-sectional | 109 | Caracas | Pediatric | · Transmission: Mostly vertical (78.89%)  · Outcomes: Viral undetectability and adherence were significantly higher in the vertical transmission group vs. the horizontal group | [94] |
| Hyperferritinemia as a prognostic factor for immunosuppression in patients with acquired immunodeficiency syndrome | Palencia L | 2011 | Cross-sectional | 40 | Zulia | Clinical behavior | · Prognostic Factor: High ferritin levels correlated with lower CD4+ counts (<200 cells/mm³) and death | [88] |
| Mortality from HIV/AIDS infection in Venezuela: 1996–2007 | Rísquez A | 2011 | Cross-sectional | 1,670 | The entire country | Clinical behavior | · Rising Mortality: HIV/AIDS deaths and rates increased significantly  · Demographics: Most affected were 25-35 year-old males  · Geographic Focus: Highest impact in Capital District and Bolívar state | [44] |
| Benefits of highly effective antiretroviral treatment in pediatric patients with HIV infection | Sabatela LS | 2011 | Longitudinal | 234 | Caracas | Pediatric | · Declining Trends: Hospitalization and mortality decreased since 2000, linked to increased HAART access  · Poor Outcomes: Linked to a lack of HAART, immunosuppression, and detectable viral loads | [93] |
| BCG vaccine-induced disease in patients with vertical infection by the human immunodeficiency virus | Siciliano L | 2011 | Cross-sectional | 96 | Caracas | Pediatric | · Complications: 16.7% experienced complications, mainly adenitis, predominantly in immunosuppressed cases  · Outcomes: High success rate (87.5%) with HAART; rare vaccine-related mortality (1.1%) | [101] |
| Assessment of nutritional status in institutionalized pediatric patients with HIV/AIDS | Villalobos D | 2011 | Cross-sectional | 60 | Zulia | Pediatric | · Key Findings: 60% had normal BMI, but also high triglycerides, low HDL-C, low hemoglobin, and inadequate calorie/nutrient intake | [97] |
| Candida species in HIV/AIDS patients with oropharyngeal candidiasis | Castrillo S | 2012 | Cross-sectional | 60 | Carabobo | Clinical behavior | · Dominant Species: *C. albicans* was the most frequent (66.6%)  · Other Species: *C. tropicalis* (13.33%) and *C. glabrata* (11.6%) were also present | [37] |
| Cardiovascular disorders in children infected with HIV at the Instituto Autónomo Hospital Universitario de Los Andes | Jiménez-Méndez MG | 2012 | Cross-sectional | 20 | Merida | Pediatric | · Key Findings: 60% had normal BMI, but also high triglycerides, low HDL-C, low hemoglobin, and inadequate calorie/nutrient intake | [95] |
| Response to highly active antiretroviral therapy in patients aged 50 years or older living with HIV/AIDS | Lugo L | 2012 | Cross-sectional | 311 | N/A | Clinical behavior | · Late Diagnosis: Over 65% of patients presented in advanced HIV stages  · Similar HAART Response: No significant difference in immunological or virological response between older (>50 yrs) and younger (<50 yrs) patients | [57] |
| Evidence of at least two introductions of HIV-1 in the Amerindian Warao population from Venezuela | Rangel HR | 2012 | Cross-sectional | 32 | The Orinoco Delta | Epidemiology | · Multiple Introductions: At least two independent HIV-1 introductions occurred  · Dissemination: Subtype B became established, while Subtype C showed no evidence of dissemination | [64] |
| Clinical and therapeutic evaluation of patients with human immunodeficiency virus (HIV) infection: infectious disease consultation | Sandoval de Mora M | 2012 | Longitudinal | 200 | Bolivar | Clinical behavior | · Demographics: Predominantly working-age adults (31-40 yrs)  · Lab/Clinical Status: Most had CD4+ counts of 201-500 cells/mm³; 28.5% had undetectable viral loads | [30] |
| Ethics and biosafety in dental care for patients with human immunodeficiency virus | Betancourt García AI | 2013 | Cross-sectional | 45 | Caracas | Miscellaneous | · Bioethical Knowledge: 40% Good, 22.2% Bad  · Biosecurity Knowledge: 26.6% Good, 48.8% Bad | [114] |
| Periodontal disease in women with HIV/AIDS | Guerra ME | 2013 | Cross-sectional | 55 | Caracas | Clinical behavior | · Immune Status: 54.4% of women had severe immunosuppression  · "IP-r" Trend: The average "IP-r" value increased with the severity of immunosuppression | [83] |
| Linfoma no hodgkin asociado a infección por virus de inmunodeficiencia humana estudio de cohorte | Hernández DE | 2013 | Longitudinal | 12 | Caracas | Clinical behavior | · Improved Response: Treatment response increased from 34% to 66% with HAART  · Mortality Shift: Cause of death shifted from opportunistic infections (pre-HAART) to lymphoma progression (post-HAART) | [112] |
| Prevalence of intestinal microsporidia and other intestinal parasites in hiv positive patients from Maracaibo, Venezuela | Rivero-Rodríguez Z | 2013 | Cross-sectional | 56 | Zulia | Clinical behavior | · Overall Prevalence: 67.86% carried intestinal parasites  · Common Protozoa: *Isospora belli* and *Blastocystis spp.* (both 17.65%)  · Microsporidia: Detected in 33.33% of samples | [32] |
| Prevalence of Encephalitozoon intestinalis and Enterocytozoon bieneusi in HIV positive patients to Maracaibo, Venezuela | Rivero-Rodríguez Z | 2013 | Cross-sectional | 50 | Zulia | Clinical behavior | · High Prevalence: Microsporidia species found in 36% of fecal samples  · Dominant Species: *Encephalitozoon intestinalis* was most frequent | [33] |
| Acquired immunodeficiency syndrome (AIDS): epidemiology and clinical features | Storino-Farina MA | 2013 | Cross-sectional | 150 | Caracas | Clinical behavior | · Top Diseases: CNS (26%) and Lung (25%) diseases were most prominent  · CD4 & Viral Load: 47% had CD4+ counts <100; 51% had viral loads <150,000 | [52] |
| Risk factors for overweight, obesity, and malnutrition in HIV-positive patients attending nutritional consultations at the center for patients with infectious diseases at the Faculty of Dentistry | Villahermosa ML | 2013 | Cross-sectional | 246 | Caracas | Clinical behavior | · Overweight/Obesity Risk: Employment was a significant risk factor | [54] |
| HIV-1 epidemic in Warao Amerindians from Venezuela: Spatial phylodynamics and epidemiological patterns | Villalba JA | 2013 | Cross-sectional | 576 | The Orinoco Delta | Epidemiology | · Prevalence: 9.55% overall, significantly higher in men (15.6%)  · Origin & Spread: Single HIV-1 subtype B introduction in the early 2000s, followed by rapid dissemination | [28] |
| Complications in dental treatments in HIV-positive children with a hematological diagnosis of anemia | Carrasco Colmenares W | 2014 | Cross-sectional | 137 | Caracas | Pediatric | · Complication Rate: 20% of children experienced infections or other complications, highlighting the need for safe, interdisciplinary care | [96] |
| Evaluation of pulmonary vascular lesions in HIV-positive patients by transbronchial biopsy | Casavilca JS | 2014 | Cross-sectional | 35 | Merida | Clinical behavior | · High Prevalence: 66.7% of patients had vascular lesions, mainly middle layer hyperplasia  · No Link to HIV Markers: Lesions were not associated with disease duration, CD4, viral load, or HAART | [74] |
| Genetic diversity of hepatitis B virus and hepatitis C virus in human immunodeficiency virus type 1-co-infected patients from Venezuela | Jaspe RC | 2014 | Cross-sectional | 512 | Caracas | Clinical behavior | · Prevalence: HBV co-infection (14%) was significantly higher than HCV (0.7%)  · Occult HBV: High prevalence (18%) in naïve co-infected patients | [77] |
| HIV-1 and GBV-C co-infection in Venezuela | Rodríguez AK | 2014 | Cross-sectional | 525 | Caracas and Lara | Clinical behavior | · Higher Prevalence: GBV-C was significantly more common in HIV-1+ patients (27%) than in HIV-1-negative individuals (11%) | [78] |
| Level of self-esteem and its relationship with the absolute value of TCD4 lymphocytes in patients living with HIV/AIDS | Ayala Rivero FJ | 2015 | Cross-sectional | 100 | Carabobo | Clinical behavior | · Self-Esteem & CD4 Count: Patients with high self-esteem had the highest average CD4 cell counts | [116] |
| The Evolving HIV-1 Epidemic in Warao Amerindians Is Dominated by an Extremely High Frequency of CXCR4-Utilizing Strains | Rangel HR | 2015 | Cross-sectional | 110 | The Orinoco Delta | Clinical behavior | · Dominant Subtype B: HIV-1 subtype B was most frequent  · Warao's Unique Reactivity: Warao Amerindians showed significantly higher reactivity to synthetic peptides than the general population | [65] |
| Fungal infections in HIV-infected patients at the Ruiz y Páez University Hospital Complex | Cermeño J | 2016 | Cross-sectional | 42 | Bolivar | Clinical behavior | · High Fungal Prevalence: 35.7% of patients had fungal infections, most commonly oropharyngeal candidiasis (60%) | [80] |
| Epidemiological characteristics and other indicators in women infected with Human Immunodeficiency Virus (HIV) in Venezuela | Carvajal A | 2016 | Cross-sectional | 300 | Caracas, Carabobo, Sucre y Merida | Epidemiology | · Diagnosis & Pregnancy Link: Recent diagnosis was significantly linked to pregnancy  · Treatment & Clinical Status: 81% received ART and 75% were asymptomatic, but 25% were in the AIDS stage | [56] |
| Short-Term Dynamic and Local Epidemiological Trends in the South American HIV-1B Epidemic | Junqueira DM | 2016 | Cross-sectional | 4,810 | South American | Epidemiology | · Regional Dominance: ~70% of HIV-1B infections occurred within the same geographic region, indicating localized epidemics | [63] |
| HIV prevalence, sexual behaviours and engagement in HIV medical care among an online sample of sexually active MSM in Venezuela | Perez-Brumer AG | 2016 | Cross-sectional | 3,175 | Territories in Latin America and the Caribbean, and in Spain and Portugal | Epidemiology | · Prevalence & Treatment: Self-reported HIV prevalence was 7.8%; 73.2% were on ART  · Risk Factors: Positive status linked to being older and having a prior STI diagnosis | [22] |
| Change of highly active antiretroviral therapy associated with adverse drug reactions in a specialized center in Venezuela | Subiela HJD | 2016 | Cross-sectional | 99 | Lara | Treatment | · High ADR Prevalence: ADRs accounted for 47.5% of first-line HAART changes  · Common ADRs & Drug: Anemia (34.3%) and hypersensitivity (20.2%) were most frequent; zidovudine was most linked to ADRs (41.4%) | [41] |
| Risk factors for HIV infection among indigenous people of the Warao ethnic group in the municipality of Antonio Díaz, Delta Amacuro State, Venezuela | De Waard J | 2017 | Cross-sectional | 150 | The Orinoco Delta | Clinical behavior | · Risk Factors: Included unstable/casual sexual partners, early sexual initiation, and lack of knowledge about HIV transmission | [29] |
| Morbidity, mortality, and failure of antiretroviral treatment in adolescents with HIV/AIDS at a referral hospital in Caracas, Venezuela | Monsalve-Arteaga L | 2017 | Longitudinal | 79 | Caracas | Clinical behavior | · Transmission: 63.2% horizontal; 36.7% vertical  · Virologic Failure: 50% experienced virologic failure after 90 months of treatment | [75] |
| Characterization of the HIV/AIDS population at the University Hospital of Caracas | Herranz ÁG | 2018 | Cross-sectional | 100 | Caracas | Clinical behavior | · Depression & Adherence: Most patients (64%) had minimal depression; treatment adherence was generally high | [39] |
| Evaluation of emerging infectious disease and the importance of SINAN for epidemiological surveillance of Venezuelans immigrants in Brazil | Lima Junior MM | 2019 | Cross-sectional | 6,252 | Brazil | Migrants | · Higher Burden: Venezuelan migrants had significantly higher rates of HIV/AIDS, leishmaniasis, and malaria than Brazilians | [8] |
| Mortality due to systemic mycoses, associated with hiv infection in venezuela. Period 1996-2013 | Lemus-Espinoza D | 2019 | Cross-sectional | 2,764 | The entire country | Clinical behavior | · Leading Causes of Death: *Pneumocystis jirovecii* pneumonia (1846 cases), followed by other mycoses (495) and candidiasis (423)  · Trends: Mortality was higher in men and peaked in the 2005-2013 period | [86] |
| Preliminary experience of pediatric patients with HIV infection treated with Dolutegravir | López MG | 2019 | Cross-sectional | 193 | Caracas | Pediatric | · DTG Use: 9.2% of patients (mostly adolescents)  · Side Effects: 70% of patients on DTG experienced no or mild adverse effects | [102] |
| Migration crisis in Venezuela: impact on HIV in Peru | Rebolledo-Ponietsky K | 2019 | Cross-sectional | 720 | Peru | Migrants | · HAART Coverage: 720 immigrant HIV/AIDS patients received HAART in Peru in 2018, mostly in Lima  · Vulnerability Factors: High vulnerability to HAART abandonment, malnutrition, and late diagnosis | [10] |
| Mortality in hospitalized patients diagnosed with human immunodeficiency virus | Pérez Vega C | 2019 | Cross-sectional | 1,322 | Venezuela | Clinical behavior | · HIV Mortality Rate: 5.52% of all hospital deaths  · AIDS-Related Deaths: 74.5% of HIV deaths were AIDS-associated | [73] |
| Prevalence of transfusion-transmissible infections in southern Lara State, Venezuela | Vizcaya-Rodríguez T | 2019 | Cross-sectional | 6,440 | Lara | Epidemiology | · Highest Prevalence: Anti-HBc (5.34%) and HBsAg (0.66%)  · Other TTIs: HIV (0.26%), HCV (0.17%), *T. cruzi* (0.42%) | [20] |
| Evaluation of high- and low-risk oncogenic human papillomavirus in the oral cavity of HIV-positive patients | Ávila M | 2020 | Cross-sectional | 31 | Caracas | Clinical behavior | · High HPV Prevalence: 61.0% of samples showed HPV infection  · Dominant Genotypes: Low-risk genotype 6 was most frequent (73.68%), followed by high-risk genotypes 18 (63.16%) and 16 (32.0%) | [45] |
| Pneumocystis jirovecii in hiv patients and suspected pneumonia: A problematic diagnosis in caracas, venezuela | Panizo MM | 2020 | Cross-sectional | 161 | Caracas | Clinical behavior | · High PCP Frequency: *P. jirovecii* detected in 76/161 samples by DIF  · Immunosuppression Link: Advanced immunosuppression increases the likelihood of *P. jirovecii* colonization | [72] |
| Clinical situation of Venezuelan migrants living with HIV in a hospital in Lima, Peru | Huerta-Vera GS | 2021 | Cross-sectional | 398 | Peru | Migrants | · Worsening Condition: Migrants arrived in worsened clinical condition over time  · ART Challenges: By late 2019, 10.8% had not started ART, and viral suppression rates (71.8%) were suboptimal | [66] |
| The influence of personality and motives on highly HIV-adherent patients from Venezuela: Theoretical and structural equation analysis | Laborín Álvarez JF | 2021 | Cross-sectional | 282 | Carabobo and Lara | Miscellaneous | · Psychological Predictors: Moderate tolerance to frustration/ambiguity and high motivation significantly predicted adherence | [111] |
| Prevalence of undiagnosed HIV in Venezuelan patients with suspected COVID-19 during the first wave: A complex syndemic | Restuccia D | 2021 | Cross-sectional | 118 | Caracas | Epidemiology | · HIV Prevalence: 4.24% of COVID-19 patients were HIV positive  · Behavioral Links: HIV-positive status linked to higher rates of homosexuality and syphilis history | [21] |
| Human cosavirus infection in hiv subjects with diarrhoea: persistent detection associated with fatal outcome | Vizzi E | 2021 | Cross-sectional | 143 | Caracas and La Guaira | Clinical behavior | · Enteric Virus Link: Enteric viruses were significantly more frequent in diarrhea cases  · HCoSV Presence: Found in 3.5% of diarrhea cases | [34] |
| Antiretroviral therapy use in selected countries in Latin America during 2013-2017: results from the Latin American Workshop in HIV Study Group | Zitko P | 2021 | Cross-sectional | 116,299 | Argentina, Bolivia, Chile, Colombia, Costa Rica, Ecuador, Guatemala, Honduras, Mexico, Panama, Peru, Dominican Republic, Uruguay, Venezuela | Treatment | · ART Shortages & Coverage: One-third of centers faced ART shortages, yet 95.1% of patients received ART by 2017 | [109] |
| Leishmania donovani and HIV co-infection in vitro: Identification and characterization of main molecular players | Maksoud S | 2022 | Experimental |  | Caracas | Clinical behavior | · Replicated Effects: Cell model replicated amplified viral/parasitic replication  · Molecular Alterations: Co-infection amplified NF-κB activation and shifted cytokine profiles from Th1 to Th2 | [91] |
| Effects of Venezuelan migration on HIV case reporting in Colombia | Molina Lubo O | 2022 | Cross-sectional | 121,503 | Colombia | Migrants | · Minor Contribution: HIV cases imported from Venezuela had a small contribution (2.26%) to the increase in cases notified in Colombia | [9] |
| Defects in immune response to Toxoplasma gondii are associated with enhanced HIV-1-related neurocognitive impairment in co-infected patients | Escobar-Guevara EE | 2023 | Experimental | 69 | Caracas | Clinical behavior | · Worsened Neurocognition: HIV-1/*T. gondii* co-infected patients showed more severe neurocognitive impairment than HIV-1 mono-infected patients | [69] |
| HBV, HCV, or syphilis coinfection with HIV in pregnant women seen at the main hospital in the capital of Venezuela | García YD | 2023 | Cross-sectional | 156 | Caracas | HIV and pregnancy | · Prenatal Care: Most (67.9%) had insufficient prenatal care despite high ART use (89.1%)  · Co-infections: HBsAg (9.3%) and syphilis (8.2%) were observed  · Outcomes: Significant rates of prematurity (18.1%) and low birth weight (17.4%) | [49] |
| Barriers and facilitators to confronting HIV/aids and syphilis experienced by Venezuelan women living in BrazilFactores facilitadores y barreras que tienen las mujeres venezolanas residentes en Brasil respecto de la infección por el VIH/sida y la sífilis] | Mocelin HJS | 2023 | Cross-sectional | 40 | Brazil | Migrants | · Barriers: Included language, costs, and the COVID-19 pandemic  · Facilitators: Included Brazil's Unified Health System and positive relationships with health professionals | [67] |
| HIV infection and engagement in the care continuum among migrants and refugees from Venezuela in Colombia: a cross-sectional, biobehavioural survey | Wirtz AL | 2023 | Cross-sectional | 6,221 | Colombia | Migrants | · Prevalence: 0.9%  · Care Continuum: Of those with HIV, 47.9% were previously diagnosed, but only 35.7% had viral suppression | [23] |
